# Use of the Internal Transcribed Spacer (ITS) Regions to Examine Symbiont Divergence and as a Diagnostic Tool for *Sodalis*-Related Bacteria

**DOI:** 10.3390/insects2040515

**Published:** 2011-11-30

**Authors:** Anna K. Snyder, Kenneth Z. Adkins, Rita V. M. Rio

**Affiliations:** Department of Biology, West Virginia University, Morgantown, WV 26506, USA; E-Mails: asnyde19@mix.wvu.edu (A.K.S.); kennethzadkins@gmail.com (K.Z.A.)

**Keywords:** internal transcribed spacer, *Sodalis*, phylogeny, evolution of symbiosis

## Abstract

Bacteria excel in most ecological niches, including insect symbioses. A cluster of bacterial symbionts, established within a broad range of insects, share high 16S rRNA similarities with the secondary symbiont of the tsetse fly (Diptera: Glossinidae), *Sodalis glossinidius*. Although 16S rRNA has proven informative towards characterization of this clade, the gene is insufficient for examining recent divergence due to selective constraints. Here, we assess the application of the internal transcribed spacer (ITS) regions, specifically the ITS^glu^ and ITS^ala,ile^, used in conjunction with 16S rRNA to enhance the phylogenetic resolution of *Sodalis*-allied bacteria. The 16S rRNA + ITS regions of *Sodalis* and allied bacteria demonstrated significant divergence and were robust towards phylogenetic resolution. A monophyletic clade of *Sodalis* isolates from tsetse species, distinct from other Enterobacteriaceae, was consistently observed suggesting diversification due to host adaptation. In contrast, the phylogenetic distribution of symbionts isolated from hippoboscid flies and various Hemiptera and Coleoptera were intertwined suggesting either horizontal transfer or a recent establishment from an environmental source. Lineage splitting of *Sodalis*-allied bacteria into symbiotic and free-living sister groups was also observed. Additionally, we propose an ITS region as a diagnostic marker for the identification of additional *Sodalis*-allied symbionts in the field. These results expand our knowledge of informative genome regions to assess genetic divergence since splitting from the last common ancestor, of this versatile insect symbiont clade that have become increasingly recognized as valuable towards our understanding of the evolution of symbiosis. These facultative and recently associated symbionts may provide a novel source of traits adaptable to the dynamic ecologies encountered by diverse host backgrounds.

## 1. Introduction

Symbioses abound in the class Insecta, where an extraordinary range of host effects, temporal and spatial distribution, and degree of co-evolution has been reported [1]. Symbioses are recognized as a widespread source of evolutionary innovation for insects. For example, insects whose diets are nutritionally unbalanced typically harbor symbionts referred to as primary symbionts (P-symbionts) that can provision essential metabolic supplementation [2], often enabling host niche expansion. P-symbiont establishment is assured through strict vertical transmission, thereby ensuring persistence of the relationship and resulting in lengthy co-evolution with its host [3,4].

Insects may also harbor facultative microbes known as secondary symbionts (S-symbionts). S-symbionts, although not obligate to host biology, may provide host benefits depending on environmental context, such as during periods of heat stress [5], parasitoid attack [6], or towards the utilization of particular host plant substrates [7]. Moreover, distantly related insects can harbor closely related bacterial S-symbionts, suggesting initial widespread microbial infection, most likely through horizontal transfer or as a free-living generalist with multiple independent host acquisitions [8]. Symbiotic establishment may then be followed by genomic tailoring through evolutionary time, leading to functional specialization complementary to host biology and ecology, similar to what has been reported with P-symbionts [9]. S-symbionts may accordingly represent intermediates in the evolutionary trajectory to an exclusively symbiotic lifestyle [10].

Tsetse flies provide ideal biological models to examine symbiosis due to the presence of a low complexity microbiota, yet representing a wide range of host-microbe relations [11]. The tsetse microbiota predominantly consists of two Gammaproteobacteria; an obligate P-symbiont *Wigglesworthia glossinidia*, and a S-symbiont *Sodalis glossinidius*, as well an Alphaproteobacteria, the facultative parasite *Wolbachia pipientis* [12]. Additionally, tsetse flies maintain significant medicinal and socioeconomic importance as the vectors of African trypanosomiasis. Consequently, symbiotic microbes are also of applied interest as their genetic manipulation offers potential disease control mechanisms [13].

In contrast to the P-symbiont *Wigglesworthia*, *Sodalis* [14] has only recently associated with the tsetse host [15]. Evidence of a recent transition into symbiosis includes its wide host tissue tropism [16], amenability towards *in vitro* culture [17], stochastic presence within tsetse field populations [18,19], and a lack of congruence with tsetse phylogeny [20]. Genomic features [21], notably; a relatively larger ∼4.2 Mb size, lack of A-T (Adenine-Thymine) bias, and the presence of phage-like and symbiosis region genes also support a recent transition into symbiosis. Despite these features, there are also some indications of *Sodalis* evolving into an endosymbiotic lifestyle such as a high proportion of pseudogenes with homologs of proteins involved in defense or in the transport and metabolism of carbohydrates and inorganic ions [21], believed to be unessential within the host. Furthermore, metabolic interplay resulting from genomic complementation between *Wigglesworthia* and *Sodalis* demonstrates early functional convergence, which may act to evade species antagonism [22].

Culture independent sequencing techniques have enabled the identification of numerous bacterial species residing within a diverse range of hosts, particularly insects [23]. One such group gaining recognition, based on high 16S rRNA gene identity, comprises *Sodalis* and related bacteria within a broad range of insects, including various Hemiptera, Diptera, Coleoptera and a Phithiraptera [24-29]. Although the 16S rRNA gene has proven quintessential in many microbial phylogenetic studies, it's exclusive use is poorly suited to differentiate recently diverged bacteria (*i.e.*, genus level and below) due to the conserved regions lacking informative characters [28,30] and the potential occurrence of homoplasy or intraspecific variation within the hypervariable regions [31].

The conserved nature of the 16S rRNA locus has lead to the use of other genome regions for the phylogenetic analyses of closely related organisms. A recent application of outer membrane genes as markers for delineating the systematics of the *Sodalis* clade demonstrated sequence variation, notably in putative surface exposed loops, likely arising from the adaptive evolution towards particular host features, such as immunity [32]. An additional example, the internal transcribed spacer regions have been shown to exhibit an accelerated evolutionary rate relative to the conventionally used 16S rRNA gene [33]. Noncoding ITS regions that separate the 16S rRNA-23S rRNA and the 23S rRNA-5S rRNA are designated as ITS1 and ITS2, respectively. Additionally, the ITS regions may encode tRNAs. Use of the ITS regions have proven informative in both sequence and length variation for the phylogenetic resolution of bacterial species [34] and strains [35].

The molecular phylogenetic analyses of bacteria, from both free-living and host-associated lifestyles, may enhance our understanding of how environmental generalists transition into symbioses that become so specialized that they rely purely on vertical transmission and are associated with the evolution of extreme genome features. In this study, we have coupled the 16S rRNA and ITS regions to examine the phylogeny and diversity of *Sodalis*-allied symbionts widely distributed throughout the class Insecta. Our results provide information on additional genetic variation among the *Sodalis*-like symbionts, further evidence to support diversification of this clade from an environmental progenitor and high likelihood for the lateral transfer of symbionts between diverse insect orders. Furthermore, we propose the ITS regions, used in conjunction with 16S rRNA, as a diagnostic tool for the identification and characterization of additional *Sodalis*-allied symbionts from insect hosts in the field. These symbionts provide snapshots of early events associated with the transitioning into insect symbiosis, and are potentially useful towards revealing both universal aspects of partner association as well as unique attributes towards particular symbioses. Methods that enhance our ability to detect these symbionts may increase the number of symbioses available for crucial comparative studies.

## 2. Experimental Section

### 2.1. Specimens and DNA Isolation

DNA was isolated from tsetse adult flies (*Glossina brevipalpis*, *G. morsitans*, *G. fuscipes*, and *G. pallidipes*), hippoboscid adult flies and pupae (*Craterina melbae*), larval stage chestnut weevils (*Curculio sikkimensis*) and *Sodalis* bacteria from *in vitro* culture following the Holmes-Bonner protocol [36]. Due to the sympatric localization of *Cu. sikkimensis* with the sister species *Cu. dentipes*, as well as the lack of distinguishable morphological features between the two species as larvae, the species identification was verified by sequencing of the mitochondrial cytochrome oxidase subunit I, *CO1* [37]. DNA samples of the ovaries of adult shieldbugs *(Eucorysses grandis)* and scutellerid stinkbugs *(Cantao ocellatus)* were obtained by using a NucleoSpin Tissue kit (Macherey-Nagel, Bethleham, PA). Additionally, the *Sodalis*-like *Biostraticola tofi* DNA, originally isolated from the biofilm of a tufa (porous rock formed by the precipitation of H_2_O) deposit [38], was obtained from DSMZ (Braunschweig, Germany). All samples were re-suspended in 1× Tris-EDTA following DNA isolation.

### 2.2. PCR Amplification and Sequencing of ITS Regions

**Figure S1:**
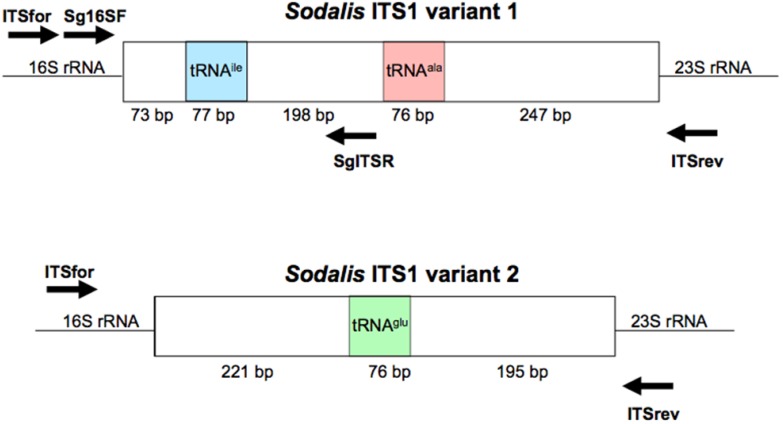
Organization of the two *Sodalis* ITS1 variants. Locations of the *Sodalis*-allied symbiont specific PCR primers (Sg16SF and SgITSR) and ITS sequencing primers (ITSfor and ITSrev) are indicated.

To amplify the ITS1 regions, primers were designed to the 3′ region of the *Sodalis* 16S rRNA gene (NC_007712; ITSfor: 5′-GGA GTG GGT TGC AAA AGA AG-3′) and the 5′ region of the 23S rRNA gene (ITSrev: 5′-CCA CCG TGT ACG CTT AGT CA-3′) (Figure S1) using the default Primer3 algorithm [39]. DNA samples were subjected to PCR amplification in 50 μL reactions consisting of 1.25 U GoTaq Flexi DNA Polymerase (Promega, Madison, WI, USA), 4 mM MgCl_2_, 1× Green GoTaq Flexi Buffer, and 0.2 mM dNTPs and primers. Amplification conditions consisted of 3 min initial denaturation at 95 °C, followed by 34 cycles of 95 °C for 30 s, 55 °C for 30 s, and 72 °C for 1.5 min, with a final elongation at 72 °C for 10 min. Negative controls were included in all reactions.

The amplification products were analyzed by agarose gel electrophoresis and viewed using Kodak 1D image analysis software. Resulting amplicons of 600–1,000 bp were extracted and purified using the QIAquick Gel Extraction Kit (Qiagen, Valencia, CA, USA). Following gel extraction, amplicons were either sequenced or ligated into pGEM-T vector (Promega, Madison, WI, USA) and transformed using *Escherichia coli* JM109 cells (Promega).

Amplicons were sequenced at the West Virginia University Department of Biology Genomics Facility with an ABI 3130 × 1 analyzer (Applied Biosystems, Foster City, CA, USA) using a 3.1 BigDye protocol (Applied Biosystems). For each DNA sample, at least three amplicons were sequenced using both forward and reverse primers and contigs were assembled using Ridom Trace Edit (RidomGmbH, Wurzburg, Germany). If any nucleotide variation was observed, 5 additional clones were subsequently sequenced to assess ITS variation.

### 2.3. Molecular Phylogenetics

Consensus sequences were created from the contigs and edited to remove the 23S rRNA regions, so that only the 16S rRNA and ITS regions were analyzed. Sequences were aligned using MUSCLE [40] and inspected and corrected manually. Percent nucleotide identity between sequences was determined using PAUP 4.0 by comparing pairwise base differences [41].

Molecular phylogenetic analyses included Neighbor joining (NJ), Maximum parsimony (MP), and Bayesian methods. NJ and MP analyses were performed using PAUP 4.0 with the Kimura's two-parameter model of nucleotide substitution and 1,000 nonparametric bootstrap (BS) replicates, as a measure of lineage support. MP heuristic searches implemented 1,000 replicates using the tree-bisection-reconnection algorithm, where starting trees for branch swapping were obtained through random Stepwise-Additions, and Max trees set at 200. Additionally, each MP analysis was performed twice, with gaps treated as either “missing data” or “5th character state”, with no differences noted among the resulting phylogenies.

Bayesian analyses were performed using MrBayes 3.1.2 [42] with Posterior Probabilities (PP) calculated. Evolutionary models to implement for each dataset where chosen using the Akaike Information Criterion in MrModelTest version 2.3 [43]. The best fit model implemented in both the 16S rRNA and ITS^glu^ or ITS^ala,ile^ analyses was the General Time Reversible + invariant sites + gamma (GTR + I + G). Additionally, Markov chain Monte Carlo parameters were set to 6 chains and 1 million generations. Stabilization of model parameters, burn-in, occurred after 800,000 generations, and every 100th tree after burn-in was used to generate a 50% majority-rule consensus tree. FigTree v1.3.1 [44] was used to construct all trees. Bold branches within trees represent incongruences between the different phylogenetic methods utilized in this study.

### 2.4. Diagnostic PCR

To explore the use of the ITS region as a diagnostic tool for *Sodalis* related bacteria, ITS^ala,ile^ nucleotide alignments were used to identify a *Sodalis* clade specific reverse primer (SgITSR 5′-ACC TTG CAT ATG CCG TCG CT-3′). This oligonucleotide can be used with the 3′ end 16S rRNA forward primer (Sg16SF 5′-TGA TTC ATG ACT GGG GTG AA-3′) (Figure S1) under the temperature profile of 95 °C for 3 min followed by 35 cycles of 95 °C for 30 s, 55 °C for 30 s and 72 °C for 30 s, with a final elongation of 72 °C for 5 min. DNA isolated (∼300 ng) from various insect hosts were subjected to the diagnostic PCR detection. Negative controls, including *E. coli* and *Bi. tofi*, were included in analyses.

### 2.5. Nucleotide Accession Numbers

The nucleotide sequences from this study have been submitted to the NCBI GenBank database. The 16S rRNA genes (and corresponding accession numbers) used in this study included; *G. brevipalpis* S-symbiont (U64870), *G. pallidipes* S-symbiont (M99060), *G. morsitans* S-symbiont (AY861701), *G. fuscipes* S-symbiont (AY861704), *Sodalis glossinidius* culture (NC_007712), *Cr. melbae* symbiont (EF174495), *Eu. grandis* S-symbiont (AB571330), *Ca. ocellatus* S-symbiont (AB541010), *Sitophilus zeamais* P-symbiont (AF548140, AF548141), *Si. oryzae* P-symbiont (AF548138, AF548139), *G. brevipalpis* P-symbiont (NC_004344), *Cu. sikkimensis* S-symbiont (AB517595), *Bi. tofi* (AM774412), *Yersinia pestis* (NC_003143), *Salmonella enterica* (NC_003198), *E. coli* (NC_000913), *Erwinia amylovora* (NC_013961), *Pantoea vagans* (NC_014562), *Vibrio fischeri* (NC_006840), *Pseudomonas aeruginosa* (NC_002516), *Bacillus cereus* (NC_004722), *Ba. subtilis* (NC_000964), and *Ba. pumilus* (NC_009848). The ITS regions (and corresponding accession numbers in the order of ITS^glu^ and ITS^ala,ile^) used in this study included; *So. glossinidius* culture (NC_007712), *Si. oryzae* P-symbiont (AF548137), *Y. pestis* (NC_003143), *Sa. enterica* (NC_003198), *E. coli* (NC_000913), *Er. amylovora* (NC_013961), *Pa. vagans* (NC_014562), *G. brevipalpis* P-symbiont (NC_004344), *V. fischeri* (NC_006840), *Si. zeamais* P-symbiont (AF548140, AF548141), *Si. oryzae* P-symbiont (AF548138, AF548139), *Ps. aeruginosa* (NC_002516), *Ba. cereus* (NC_004722), *Ba. subtilis* (NC_000964), and *Ba. pumilus* (NC_009848).

## 3. Results and Discussion

### 3.1. Amplification of ITS Regions

**Figure S2:**
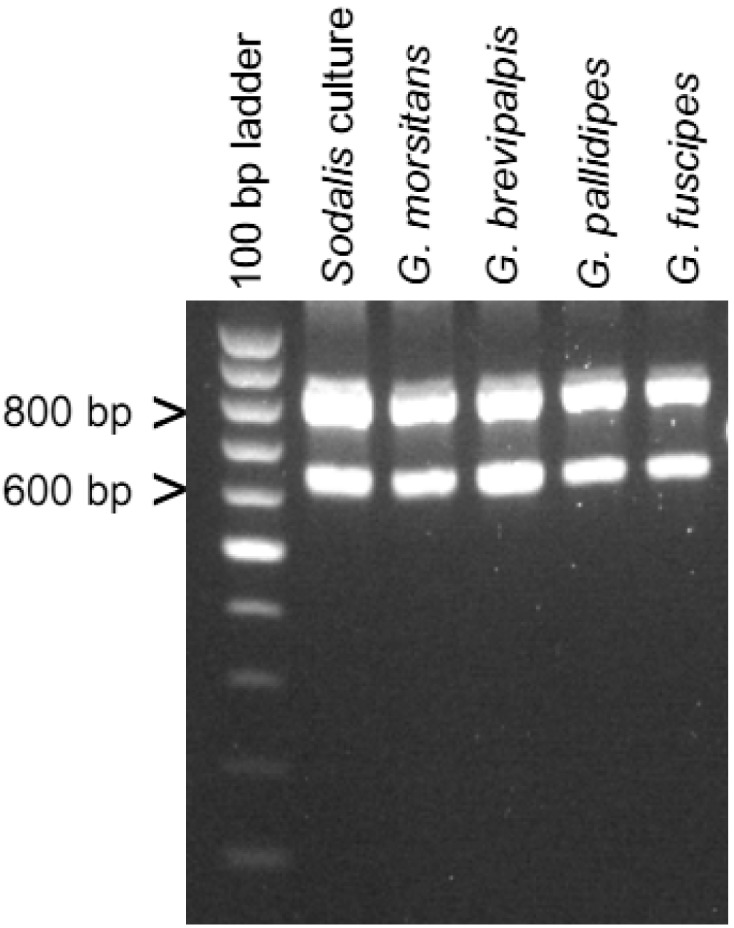
Specificity of ITS primers for *Sodalis*. The ITSfor and ITSrev primers do not amplify the corresponding *Wigglesworthia* ITSglu. Both *Sodalis* ITS1 variants were amplified; the 646 bp amplicon contains the 492 bp ITSglu, while the 825 bp amplicon contains the 671 bp ITSala,ile. Tsetse host species are indicated.

The annotated *Sodalis* genome contains 2 distinct ITS1 regions [45]; a 671 bp ITS which encodes both tRNA-ala and tRNA-ile (ITS^ala,ile^) and an additional 492 bp ITS region containing tRNA-glu (ITS^glu^). Although multiple copies are found throughout the genome, no sequence divergence is observed within ITS regions due to the pervasiveness of concerted evolution in the rRNA operon [45,46]. In contrast, the genome of the *G. brevipalpis* [47] and *G. morsitans* [48] P-symbiont *Wigglesworthia* retains only two copies of an ITS1 region encoding only tRNA-glu (ITS^glu^), consisting of 270 bp or 225 bp, respectively, with no intragenomic nucleotide sequence variation and an intergenomic nucleotide sequence identity of 63.7%. The primers used in this study were designed to be specific to *Sodalis* and did not amplify the *Wigglesworthia* ITS region (Figure S2).

Upon sequencing of the ITS regions, ranges in both size (Table 1) and intra- and inter-genomic variation (Table 2) were observed in both ITS^ala,ile^ and ITS^glu^ regions for the examined microbes. Interestingly, the chestnut weevil *Cu. sikkimensis* S-symbiont isolate only amplified one PCR product, with an ITS^ala,ile^ not detected. ITS variation has been linked to functional divergence and differences in ecological capabilities in bacteria [49-51], whether the lack of amplification of the ITS^ala,ile^ from the *Cu. sikkimensis* S-symbiont represents an adaptive response to particularities of that symbiotic lifestyle remains unclear. Lastly, the free-living *Bi. tofi* amplified two distinct intragenomic ITS^glu^ regions with the highest intragenomic diversity (86.1%–87.5%) observed within this study (Table 2). The amplification of two distinct ITS^glu^ regions by the free-living *Bi. tofi* may represent variation found in the ancestral lineage, which has been purged within the symbionts. In support, *E. coli* also exhibits a similar trend by encoding four ITS^glu^ copies within its genome, which can be divided into two groups, ranging in nucleotide sequence identity from 88.2%–99.2%. It is also tempting to note that *Bi. tofi* was isolated from the biofilm of a tufa deposit [38] which would have increased exposure to the introduction of foreign DNA, potentially contributing to ITS^glu^ variation. Contrastingly, horizontal transfer events are thought to be negligible in the evolution of endosymbionts due to their intracellular localization and reduced recombination rates [10].

### 3.2. ITS Sequence Variation and Molecular Systematics of Sodalis-Allied Symbionts

The ITS sequences, originating from insects harboring *Sodalis* and allied bacteria, were subject to molecular phylogenetic analyses. When examining the ITS^glu^ and ITS^ala,ile^ regions, there was a range of conservation throughout the sequences. Due to functional constraint associated with the tRNA genes, *Sodalis* and related bacterial sequences shared close to 100% sequence identity, with the exception of a low number of point mutations (*i.e.*, <5 between different isolates). Additional conserved motifs, within both ITS regions, were the box A anti-terminator sequence for RNA transcription [52], where all *Sodalis* and related bacteria encoded an identical sequence (5′-CGCTCTTTAACAAT-3′) and the RNAse III recognition sites located proximal to the 3′ end of the 16S rRNA gene and the 5′ end of the 23S rRNA gene [53].

**Figure 1:**
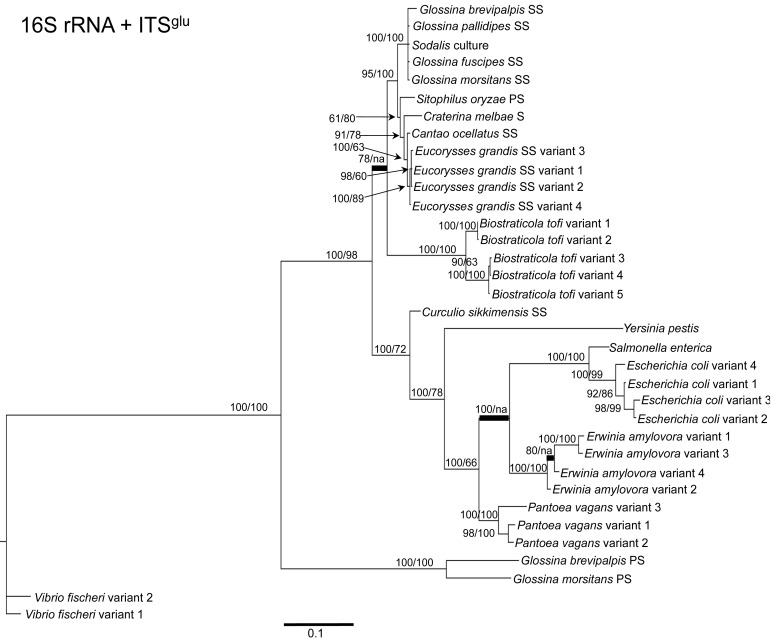
Phylogenetic placement of *Sodalis* and related symbiotic bacteria within Gammaproteobacteria based on 16S rRNA and ITS^glu^ concatenation. A Bayesian tree, inferred from a total of 2,467 unambiguously aligned nucleotide sites, with support values indicating Bayesian posterior probabilities (PP)/MP bootstrap (BS) is shown. PP indicated as percentage, *i.e.*, PP = 0.95 is depicted as 95. Branches constrained with MP are shown in bold. For insect symbionts, host species are indicated. S = symbiont, SS = S-symbiont, PS = P-symbiont. Scale bar represents substitutions/site.

**Figure 2:**
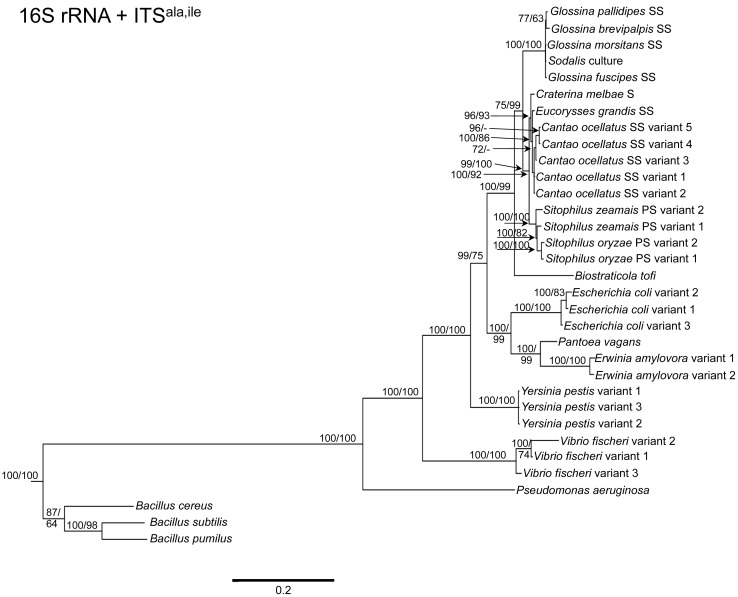
Phylogenetic placement of *Sodalis* and related symbiotic bacteria within Bacteria based on ITS^ala,ile^. A Bayesian tree, inferred from a total of 2,762 unambiguously aligned nucleotide sites, with support values indicating Bayesian posterior probabilities (PP)/MP bootstrap (BS) is shown. PP indicated as percentage, *i.e.*, PP = 0.95 is depicted as 95. Branches collapsed with Bayesian analysis are shown in bold. NJ analysis resulted in a similar phylogeny. For insect symbionts, host species are indicated. S = symbiont, SS = S-symbiont, PS = P-symbiont. Scale bar represents substitutions/site.

To determine the utility of the 16S rRNA + ITS regions as a tool for resolving relationships and understanding the degree of diversity between *Sodalis* and allied symbionts, NJ, MP and Bayesian phylogenetic analyses were performed. The resulting phylogenetic trees of 16S rRNA + ITS^glu^ and 16S rRNA + ITS^ala,ile^ (Figures 1 and 2, respectively) gave substantially the same topology and were generally concordant with 16S rRNA based phylogeny [29,32], yet provided stronger resolution among the *Sodalis* and allied bacteria as indicated with relatively higher MP bootstrap (BS) and Bayesian posterior probability (PP) support for most nodes. Phylogenetic analyses of ITS based trees reflect the conserved nature of ITS regions within tsetse isolates (Figures 1 and 2), with both ITS^glu^ and ITS^ala,ile^ trees containing a well-supported monophyletic nest of *Sodalis* isolates, distinct from other *Enterobacteriaceae*, and suggestive of diversification potentially attributed to tsetse host adaptation. Increased sequence divergence of ITS^ala,ile^ with *Sodalis* isolates from *G. pallidipes* and *G. brevipalpis* hosts was also observed, although BS and PP values were not robust at this node.

Within the *Sodalis*-like symbiont clade, the *Sitophilus* P-symbiont ITS^ala,ile^ sequences also displayed significant variation from the remaining insect symbiont sequences, resulting in their own clade with high MP BS and Bayesian PP support (Figure 2). Contrastingly, 16S rRNA based phylogenies intertwine the symbionts from various *Sitophilus* hosts [32], due to rRNA heterogeneities within a genome, most likely arising from a reduction in the efficacy of recombinational gene conversion due to the loss of associated DNA repair loci [54]. Moreover, *Cr. melbae*, *Eu. grandis*, and *Ca. ocellatus* symbionts group together with high support, in both phylogenies despite being housed in insects of two different taxonomic orders, suggesting a recent establishment within each host from a common environmental progenitor and/or possible horizontal transfer of symbionts. The infection of *Sodalis*-like bacteria has been reported from only a minority of populations with low frequency in both *Ca. ocellatus* [24] and *Eu. grandis* [29], this erratic distribution further supports relatively recent host establishments. Displaying similarities in their infection patterns, the aphid S-symbionts *Candidatus* Hamiltonella defensa and *Candidatus* Regiella insecticola have been shown to establish within phylogenetically diverse hosts [55]. A similar phylogenetic pattern has also been described for the monophyletic *Arsenophonus* genus where some of the symbionts display parallel evolution with their hosts while others demonstrate haphazard association with distant host taxa ranging from insects to plants [30]. Furthermore, the internal node depicting the most recent common ancestor of *Bi. tofi* and the *Sodalis*-allied bacteria, within both the 16S rRNA + ITS^glu^ and 16S rRNA + ITS^ala,ile^ phylogenies, represents inferred lineage splitting that gives rise to symbiotic and free-living sister groups. The transition into symbiosis by the *Sodalis*-allied bacteria appears to have occurred following the diversification of the environmental *Bi. tofi*. Lastly, combining both 16S rRNA and ITS^glu^ regions in our molecular phylogenetic analyses, proved useful towards resolving the taxonomic placement of the *Cu. sikkimensis* S-symbiont. Previously, the phylogenetic placement of this symbiont, based on either the 16S rRNA [24,26,32] or the *groEL* [26] gene, had remained uncertain with low support for grouping with *Sodalis*. Upon utilizing both 16S rRNA and ITS^glu^ regions, the *Cu. sikkimensis* S-symbiont lineage was placed outside of the *Sodalis*-allied symbiont/*Bi. tofi* clade with strong statistical support (Figure 1).

### 3.3. Diagnostic PCR Detection of Sodalis-Like Symbiotic Bacteria

**Figure 3:**
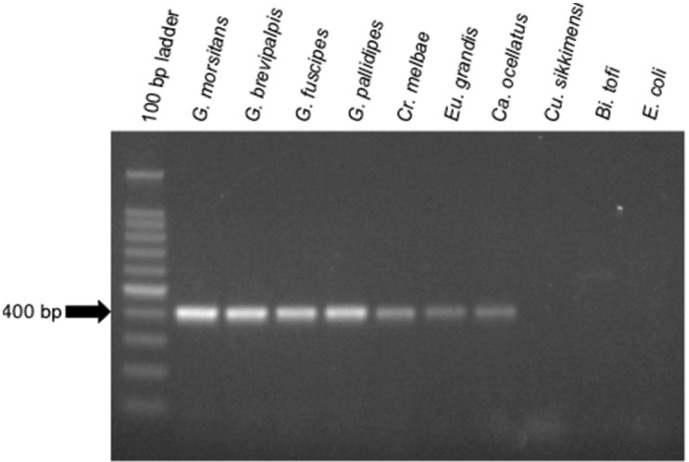
Diagnostic PCR detection of *Sodalis* and allied insect symbionts using ITS^ala,ile^ specific oligonucleotides and 300 ng of DNA template. An approximately 400 bp product was amplified. Lanes are labeled by either insect host or culture isolate (*i.e.*, *Bi. tofi* and *E. coli*).

To aid in the detection of *Sodalis*-allied bacteria in novel insect hosts, clade specific ITS primers were synthesized. Using this primer set, with the exception of *Cu. sikkimensis* which appears not to encode an ITS^ala,ile^ region, amplicons were consistently detected in all insect hosts from this study (Figure 3). This primer set was specific to symbiotic *Sodalis*-allied bacteria and did not amplify the free-living relative *Bi. tofi*, *Cu. sikkimensis* S-symbiont, and *E. coli* isolates. We propose the use of this oligonucleotide set as a diagnostic marker for the identification of additional *Sodalis*-allied symbionts in the field.

### 3.4. Potential Implications for Host Acquisition by Symbionts

Symbiosis is a significant component in the ecology of many microbes and insects in nature. Likewise, the origins of bacterial symbioses are tremendously diverse, ranging from evolutionary transitions between various host associations and environmental lifestyles [56]. Our results support that the infection of *Sodalis*-like bacteria have evolved repeatedly, through multiple opportunities, in a wide array of insect lineages. High nucleotide similarity in the ITS regions among isolates from diverse insect hosts (*i.e.*, hippoboscid, shieldbug and stinkbugs) may suggest horizontal transfer among insect species, or establishment by a free-living generalist with an enhanced capability to infect a broad range of insect hosts coupled with insufficient time for diversification. Other symbionts specifically, *Sodalis* and *Sitophilus* symbionts within tsetse and weevil hosts respectively, demonstrate clear separation from other Enterobacteriaceae indicating sufficient association time to allow for diversification of the examined ITS regions. Symbionts of recent origin are believed to be potential sources of novel traits, contrary to P-symbionts which are incapable of such due to genome degradation and secluded host intracellular localization (conferring protection from host immunological defenses but also shielding these microbes from acquiring new genes through horizontal transfer) resulting from extensive host co-evolution.

We speculate that the radiation of *Sodalis*-like bacteria into a diverse range of insects may follow the evolutionary source-sink model [57]. This model illustrates possible events in the early and intermediate stages of establishment into novel habitats, where an evolutionarily stable reservoir (*i.e.*, source), has members that migrate from the population into relatively unstable habitats (*i.e.*, sinks). Once in a sink, the population faces new challenges, such as host immune defenses or competition with resident microorganisms. In some cases, continuous emigration from the reservoir may enable adaptive evolution within the population and possibly transform the sink into a new source, able to persist and maintain throughout generations of its host, as a self-sustaining population. The symbiotic association of *Sodalis* with tsetse may be an example of a sink that has evolved into a source, whereby symbiont localization in the milk glands [58,59] (an organ used to feed tsetse larval instars during in utero development), now ensures vertical transmission to future generations of tsetse hosts. The source-sink model of evolution, although traditionally associated with pathogen emergence [60,61], may also prove beneficial towards our discussion on the evolution of symbiosis. Additional studies are needed to demonstrate if positive population growth persists through host reproduction in other insect hosts and to determine the mechanisms enabling symbiont transmission.

The recent discoveries within diverse insects of bacteria closely related to *Sodalis*, raises many experimentally approachable questions, with arguably the most significant being the characterization of conferred benefits and contributory roles towards host phenotypes. The molecular diagnostic markers proposed in this study will facilitate additional identification of related microbes in novel hosts, which will increase the number of symbioses available for comparative genomic and functional studies that aim to elucidate the reciprocal adaptations arising from symbiosis. By integrating into different host backgrounds, the outcomes of the symbioses are likely to not only be varied, but also significantly affect both partners due to tailoring in response to differences in host ecology and physiology.

## 4. Conclusions

This study reports the utility of the ITS region as a tool for both identification and enhanced resolution of the diversity associated with the ever increasing *Sodalis* allied insect symbiont clade. The similar ITS sequences observed among the tsetse *Sodalis* isolates support previous research describing its lack of divergence between tsetse species [20,32,62]. Importantly, the ITS genomic regions were able to further resolve the relatedness of *Sodalis*-allied bacteria and group the insect host associated bacteria distinct from environmental relatives, providing evidence for its use in future investigations. Utilizing genomic regions, such as surface encoding genes, which may evolve to adapt to specific host backgrounds [32,63], along with ITS regions, with its increased evolutionary rate in comparison to the adjacent 16S rRNA gene [33], as tools to understand the evolution of and ecological adaptations made by symbiotic bacteria will enhance the understanding of steps during symbiont transition. Additionally, the use of *Sodalis*-clade specific primers described in this study provides a diagnostic tool that will aid in the rapid detection of members of this group in field studies within novel insect hosts, further facilitating comparative studies which aim to characterize the reciprocal adaptations involved in different symbioses.

## Figures and Tables

**Table 1:** Comparison of the ITS^glu^ and ITS^ala,ile^ lengths for *Sodalis* and allied symbionts. With the exception of *Sodalis* from culture, host species are indicated.

	**ITS^glu^**	**ITS^ala,ile^**
	**Length (bp)**	**Length (bp)**
*G. brevipalpis* SS	492	671
*G. fuscipes* SS	492	671
*G. pallidipes* SS	492	671
*G. morsitans* SS	492	671
*Sodalis* culture	492	671
*Si. oryzae* PS	668	836, 837
*Si. zeamais* PS	n/a	837
*Cr. melbae* S	544	861
*Ca. ocellatus* SS	693	861
*Eu. grandis* SS	463	861
*Cu. sikkimensis* SS	307	Not detected
*Bi. tofi* (tufa deposit)	604, 614	796

S: symbiont; PS: primary symbiont; SS: secondary symbiont.

**Table 2:** Percent nucleotide identity of ITS regions among *Sodalis* and allied symbionts. Host genera are specified, with the exception of the free-living *Biostraticola* genus.

**ITS^glu^ % identity**							
*Glossina*	*Sitophilus*	*Curculio*	*Craterina*	*Cantao*	*Eucorysses*	*Biostraticola*	
98.6–100	94.6–96.0	69.2–69.8	93.3–93.9	94.6–96.0	94.5–96.7	76.6–79.2	*Glossina*
	100	68.9	90.8	93.7	96.8–97.5	73.9–75.1	*Sitophilus*
		100	68.8	70.3	70.5–71.2	67.1–68.0	*Curculio*
			100	95.3	93.6–94.2	74.2–79.2	*Craterina*
				100	98.7–99.4	73.0–76.8	*Cantao*
					99.4–100	79.8–81.3	*Eucorysses*
						86.1–100	*Biostraticola*
**ITITS^ala,ile^ % identity**					
*Glossina*	99.4–100				
*Sitophilus*	86.3–87.3	97.5–100			
*Craterina*	87.0–87.7	94.9–95.7	100		
*Cantao*	84.6–87.2	94.4–97.8	98.0–99.1	99.3–100	
*Eucorysses*	86.8–87.3	94.7–95.4	98.5	98.7–99.3	100
*Biostraticola*	74.6–75.1	73.6–73.9	75.0	68.4–74.9	74.4	100
	*Glossina*	*Sitophilus*	*Craterina*	*Cantao*	*Eucorysses*	*Biostraticola*
